# Seeded growth of ultrathin gold nanoshells using polymer additives and microwave radiation

**DOI:** 10.1038/s41598-021-97171-0

**Published:** 2021-09-08

**Authors:** Laurent Lermusiaux, Marie Plissonneau, Laure Bertry, Glenna L. Drisko, Valérie Buissette, Thierry Le Mercier, Etienne Duguet, Mona Tréguer-Delapierre

**Affiliations:** 1 Univ. Bordeaux, CNRS, Bordeaux INP, ICMCB, UMR 5026, 33600 Pessac, France; 2 Solvay R&I, 52 rue de la Haie Coq, 93306 Aubervilliers, France; 3 Present Address: Laboratoire de Chimie, CNRS, Ecole Normale Supérieure de Lyon, 46 allée d’Italie, 69364 Lyon, France

**Keywords:** Nanoparticles, Synthesis and processing

## Abstract

Nanoshells made of a silica core and a gold shell possess an optical response that is sensitive to nanometer-scale variations in shell thickness. The exponential red shift of the plasmon resonance with decreasing shell thickness makes ultrathin nanoshells (less than 10 nm) particularly interesting for broad and tuneable ranges of optical properties. Nanoshells are generally synthesised by coating gold onto seed-covered silica particles, producing continuous shells with a lower limit of 15 nm, due to an inhomogeneous droplet formation on the silica surface during the seed regrowth. In this paper, we investigate the effects of three variations of the synthesis protocol to favour ultrathin nanoshells: seed density, polymer additives and microwave treatment. We first maximised gold seed density around the silica core, but surprisingly its effect is limited. However, we found that the addition of polyvinylpyrrolidone during the shell synthesis leads to higher homogeneity and a thinner shell and that a post-synthetic thermal treatment using microwaves can further smooth the particle surface. This study brings new insights into the synthesis of metallic nanoshells, pushing the limits of ultrathin shell synthesis.

## Introduction

Metallic nanoshells (or core@shell particles) are nanoparticles made of a dielectric core surrounded by a metallic coating. They have attracted much attention over the past two decades because of their intense optical properties. Independent control over two parameters, i.e. the core radius and shell thickness, offers good tunability over the absorption and scattering cross-sections of the particles in the visible and near-infrared region. Therefore, they have been used for many applications such as water desalination and purification, as well as sterilisation^1^, fluorescence enhancement of dye molecules^2^, improvement of dye-functionalised solar cell^3^, and biosensing^4^. In general, the optical extinction of metallic nanoshells red shifts as shell thickness decreases and is located mostly in the near-infrared region for thicknesses below 10 nm, making particles with ultrathin shells particularly interesting for biomedical applications in the near-infrared window^5,6^.

The synthesis of gold nanoshells was first described by Oldenburg et al*.* in three steps: surface amination of silica cores, deposition of Duff’s seeds (2–3 nm gold seed) and regrowth of the seeds using a gold plating solution and a reducing agent^7^. The latter is preferentially formaldehyde because it favours a homogeneous seed growth as compared to sodium borohydride or hydroxylamine hydrochloride^8^, and because it is more practical than gaseous CO^9,10^. In terms of the synthesis mechanism, the growing seeds eventually touch and merge to form a shell. Previous experiments have shown a qualitative relationship between the seed density and the shell thickness: an increase in seed density produces thinner shells^10,11^. However, a quantitative description of this relationship is not straightforward. The formation of a continuous shell results not only from gold plating, but also from an additional process involving seeds coalescing into a smaller number of particles on the surface in the form of droplets^12^. The seed coalescing process is ascribed to Ostwald ripening, resulting from attractive Van der Waals forces between neighbouring seeds. This leads to a commonly observed intermediate state (between particles decorated with seeds and a continuous nanoshells) for which particles have a raspberry-like shape^7,8,13^ and whose optical properties are well described by numerical simulations^12^. After regrowth, the synthesis eventually yields continuous shells having at least 15 nm of thickness^2^. Unfortunately, only by getting a thinner continuous shell can the red shifted extinction spectra be obtained.

Since the first report of nanoshells^7^, many variations to the protocol have been proposed to improve or accelerate the synthesis. The most common modifications include adapting the functionalising agent, the reducing agent and the reaction conditions^11,14–18^. It appears that many parameters have direct effects on the synthesis products, such as smoothness, homogeneity and continuity of the metallic shell, yet few enable the synthesis of an ultrathin continuous shell. For example, the quality of the metallic shell is dependent on the age of the seed solution^19,20^ and the gold plating solution^21^, the pH^22^ of the latter, as well as the stirring speed during the formation of the shell^13^. Ultrathin shells have so far been obtained through a combination of specific parameters, such as high seed density and using CO_(g)_ as the reducing agent^10^. Because of the high sensitivity of nanoshell synthesis to numerous parameters, to study the effects of a given parameter, it is primordial to synthesise reference samples under the same reaction conditions.

In this paper, we produce nanoshells using a seeded-growth process, varying three independent parameters to study how they improve the synthesis of ultrathin shells: seed density, polymer additives and microwave treatment. We first demonstrate that, surprisingly, maximising gold seed density on silica particles does not help to produce ultrathin shells. The seed density on the silica particles is altered by increasing the ionic strength during the seed deposition and performing a second functionalisation/deposition step. Although the change in seed density significantly impacts the optical spectra before shell growth, after growth, the spectra are virtually identical. We then study adding a polymer during shell growth to favour the formation of a thin shell. Finally, we study flash microwave radiation treatment as a post-synthetic tool to smooth the nanoshells. Compared to the reference samples, these two innovative approaches led to thinner and smoother shells, and exhibited an increased intensity in the extinction spectra in the near infrared.

## Results and discussion

### Seed-covered silica particles

**Figure 1:**
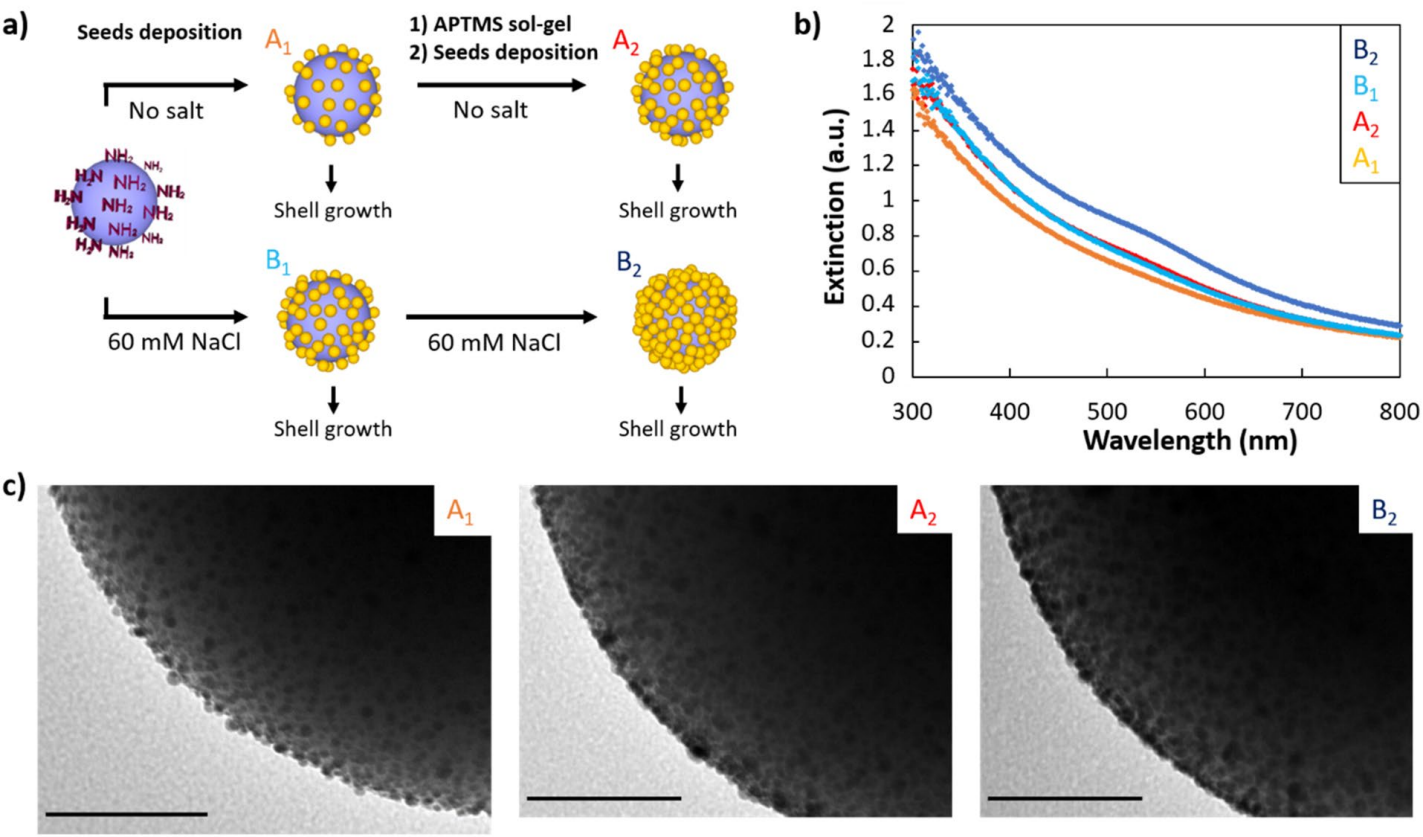
Silica particles covered with different gold seed densities. (**a**) Scheme of the different parameters used during the seed deposition onto APTMS-functionalised silica particles. The four samples produced are then used to grow core@shell particles. (**b**) UV–Vis spectroscopy of the four different seed-covered silica particle samples. (**c**) TEM images of the samples corresponding to the three different extinction curves. Scale bars represent 50 nm.

In order to study the effect of seed density on the synthesis of ultrathin gold nanoshells, we synthesised nanoshells using silica particles covered with different seed densities. We used and combined two different approaches to vary and maximise the seed density on the silica particles. Because the nanoshell synthesis is highly sensitive to different experimental conditions, the different samples were prepared in parallel, to minimise the effect of environmental parameters. We first pre-synthesised highly monodisperse (polydispersity index less than 2%), ultra-smooth 320 nm silica spheres using a step-by-step growth^24^. Monodispersity improves the ensemble optical properties^25^. After functionalising the silica particles with amine groups by surface condensation of (3-aminopropyl)trimethoxysilane (APTMS), they were mixed with 14-day old seeds under different conditions (Fig. 1a). First, half were mixed without NaCl and half at a salt concentration of 60 mM of NaCl (samples A_1_ and B_1_ in Fig. 1a). Salt decreases the electrostatic repulsion between the particles enabling a higher seed deposition density^11^. UV–Vis spectroscopy confirms the increased density, showing a stronger extinction resulting from a larger number of gold seeds in the sample, the silica particle concentrations being equal (Fig. 1b). Half of these batches were used to grow a nanoshell and the two other halves were then functionalised a second time, following the protocol described in a paper reporting a seed coverage increase from 30 to 60%^10^. In this step, pre-polymerized APTMS is used as a linker to reactivate the first gold-seeded silica core. The seed deposition was then performed without NaCl (sample A_2_, synthesised from A_1_) or with a salt concentration of 60 mM (sample B_2_, synthesised from B_1_). We observe that the UV–Vis spectrum of A_2_ and B_1_ are nearly overlaid meaning that the seed density is very close in both samples (Fig. 1b). It demonstrates that performing a double functionalisation/deposition step is roughly equivalent in seed density to a single deposition using 60 mM NaCl. However, the highest density is obtained for the sample that had both treatments: double functionalisation/deposition and salt used during the deposition, as confirmed by UV–Vis and TEM images (Fig. 1b,c and Fig. S1). In the electron microscopy images, the seed density difference is especially visible on the edge of the particles, where we can observe individual isolated seeds for the sample with the lowest density, and a tight seed packing for the sample with the highest density. However, given the low contrast between the seeds and the silica, and the intensity change between different electron microscopy images, it is extremely difficult to provide a precise seed coverage for all the samples. We estimate coverage to be around 30–40% for A1, 50–60% for A2 and B1, and 60–70% for B2, based on zoomed-in dark field TEM images (Fig. 1c and Fig. S1) as performed in previous reports^10,26^.

### Nanoshell synthesis

**Figure 2:**
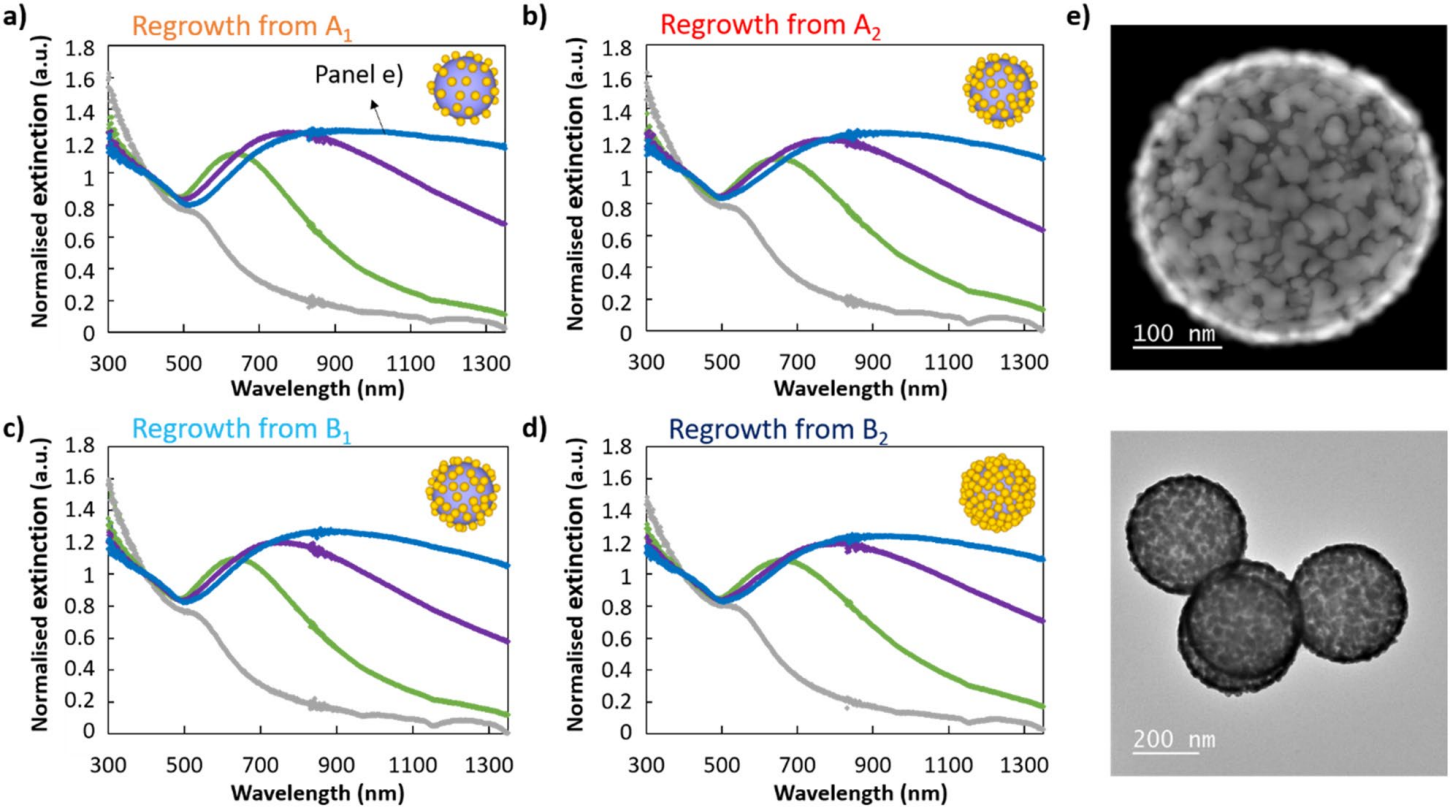
Core@shell optical properties. UV–Vis spectroscopy of the silica particle samples—(**a**) A_1_, (**b**) A_2_, (**c**) B_1_ and (**d**) B_2_—after re-growth of the gold seeds with four identical gold plating solution quantities, namely a concentration of 0.02 (grey), 0.16 (green), 0.32 (purple) and 0.53 (blue) mM. (**e**) (Top) High-angle annular dark-field and (bottom) bright-field TEM images of the sample shown in blue plot in panel (**a**).

**Figure 3:**
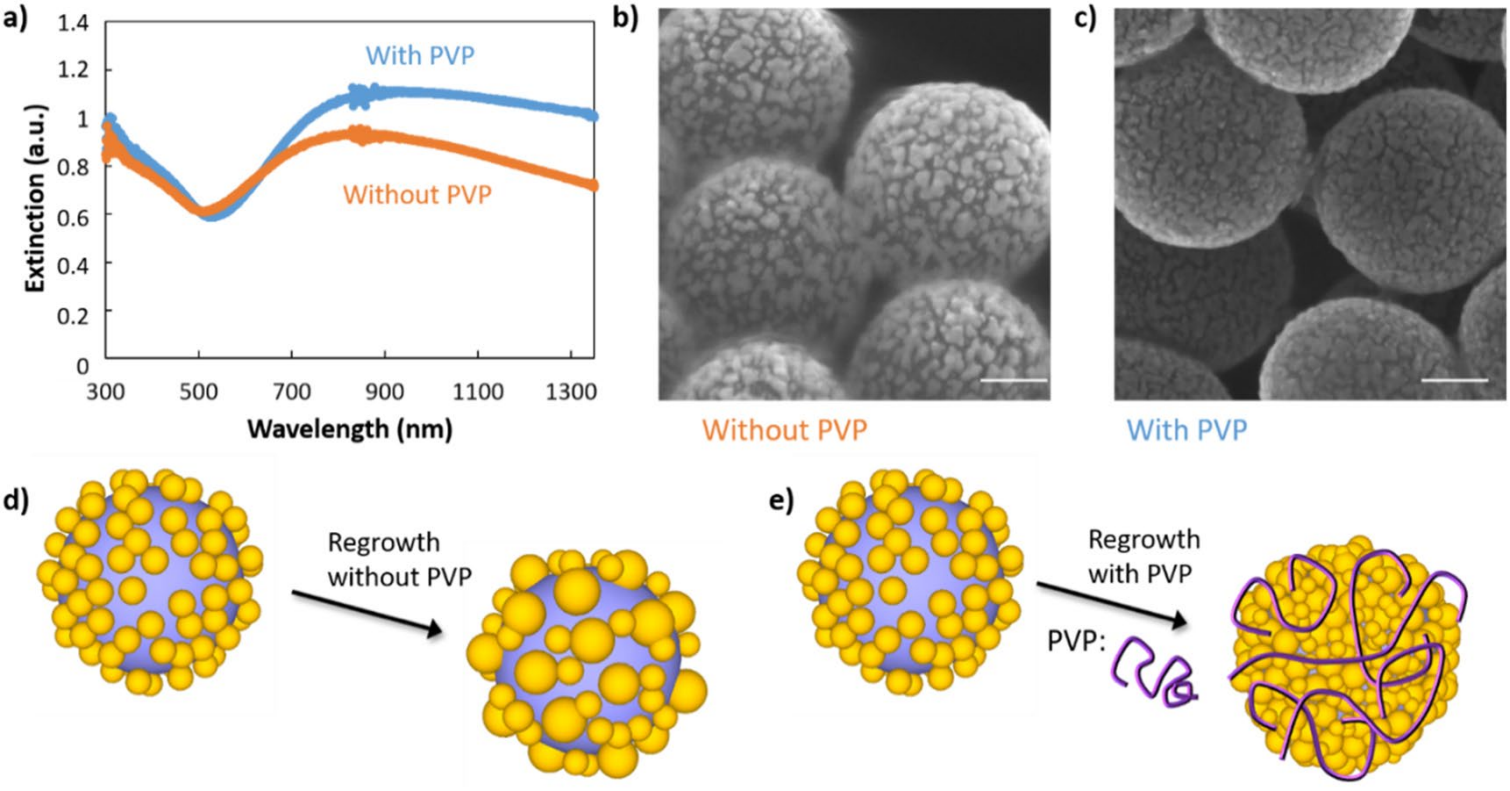
PVP effect on the synthesis of thin shells. (**a**) UV–Vis spectroscopy and SEM images of core@shell particles synthesised (**b**) without and (**c**) with PVP. The scale represents 100 nm. Synthetic representation of gold nanoshell formation when the regrowth of Au seeds is performed (**d**) without and (**e**) with PVP.

To study the effect of the seed density on the synthesis of ultrathin shells, the four previous samples, which have the same silica particle concentration and three different seed densities, were used to grow a metallic shell using a gold plating solution and formaldehyde as a reducing agent. The nanoshell synthesis was performed using aliquots of the four samples of seed-covered particles, which were mixed with different quantities of gold plating solution (100, 800, 1600 and 2666 µL, corresponding to gold salt concentrations of 0.02, 0.16, 0.32 and 0.53 mM, respectively). The optical properties of the different particles produced were measured by UV–Vis spectroscopy (Fig. 2a–d). For the lowest gold plating solution volume, we observe a single peak around 520 nm, corresponding to the plasmon resonance of small gold particles. This peak strongly red shifts and intensifies with increasing gold concentration, resulting from both the particle size increase and the plasmon coupling between neighbouring gold particles^12^. This intermediate state corresponds to the formation of gold droplets on the silica particles surface, resembling raspberry-like particles (as presented in Fig. 3d)^7,8,12,13^, instead of a uniform growth of seeds. The origin of this uneven particle growth is not completely clear as several phenomena might occur. Steric hindrance could explain this behaviour: the largest surface-bound seeds probably grow faster, as they have a larger reactive surface protruding farther out of the silica surface. As they grow, it becomes even more difficult for small seeds on the surface to react, therefore favouring the continuous and selective growth of the larger particles, which eventually dewet to minimize their surface energy. Alternatively, Preston et al. noted that some areas initially containing small seeds were later bare^12^, indicating that droplets are formed from several seeds. This decrease in the number of seeds could also result from particle detachment during the regrowth, observed, for example, when using very fresh gold seeds or from seeds not covalently bound to the silica surface^27^. It could also result from the increase of the attractive Van der Waals forces between neighbouring seeds during the seeds growth, forcing some of them to detach to form aggregates. Overall, this behaviour results from various mechanisms and prevents the formation of ultrathin shells. Particles produced with the highest gold plating solution volumes exhibit an increase in extinction in the near-infrared region, corresponding to droplets merging and forming a percolated shell. High-resolution TEM images of the largest particles show a shell of approximately 13 nm on average, mostly continuous but not smooth and exhibiting faults, evidencing the formation of large droplet (Fig. 2e and Fig. S2, Table S1). The shape of the extinction spectra is quite typical^10,12^ and differs from numerical simulations, which exhibit well-defined peaks of smaller width. The difference might result from roughness and discontinuities in the metallic shell^11,13,22,28^ leading to a red shift and broadened resonance^29^. Good correlation between the numerical simulations of the particle extinction and the measured UV–Vis spectrum is obtained for large shell thicknesses (> 20 nm) as the defect contribution to the optical response diminishes^30^.

Surprisingly, particles made from different seed densities possess very similar optical properties after shell growth (Fig. 2a–d). Because of the strong dependence of the optical properties of nanoshells on the shell thickness^7^, this means that all samples are nearly identical. In other words, it demonstrates that the relationship between the seed density and the achievable shell thickness is more complex than originally thought. The decrease of the shell thickness reaches a plateau when the seed density is higher than 30–40% (value estimated for the sample A_1_ made in a single deposition step and no salt^10,26^). We assume that this behaviour originates from the formation of the droplets, which must be relatively insensitive to the seed density above a certain threshold. This lack of effect from seed density is different to what was observed in previous papers^10,11^, however, it could be accounted for by other parameters. In Zhang and coworkers’ paper^10^, the use of CO as reducing agent may produce a different formation mechanism, thus seed coverage density may impact shell thickness with this reducing agent. In García-Soto and coworker’s article^11^, differences with our protocol regarding a longer seed deposition process and the seeded-silica particle ageing, could explain the behavioural difference. We thus pursued alternative methods to decrease shell thickness.

#### Comparison of the shell formation mechanism using polyvinylpyrrolidone

Based on our previous conclusions, our aim was to find an approach that minimises the formation of large gold droplets during the nanoshell synthesis, in order to achieve an ultrathin shell. It has been shown that the use of polymers, polyvinylpyrrolidone (PVP) or polyethylenimine, could affect the quality and shell thickness in the synthesis of metallic nanoshells^23,31,32^. For example, polyethylenimine has been used as a shell growth facilitator and to regulate the reaction rate, favouring the formation of a thin and continuous silver shell of about 10 nm^23^. Here, we investigated this strategy and studied the effect of polyvinylpyrrolidone on the growth of gold nanoshells. PVP is one the most widely used steric agents and exhibits a good affinity for both gold and silica, due to its amphiphilic character. When mixed with core–shell particles, it adsorbs onto the surfaces to form a layer whose density and thickness depend on the polymer molecular weight^31–36^. In our experiment, PVP (40 kg/mol) was added to our seeded silica particles (sample B_1_) before the regrowth of the seeds. The thickness of the PVP layer of such length is difficult to estimate, likely below 10 nm.

The effect of PVP on the synthesis of thin nanoshells was studied by simultaneously preparing two samples, one with and one without PVP. First, we observed that upon addition of PVP to the solution, the gold regrowth reaction rate is strongly affected, with a reaction time increasing from a few minutes to a couple of hours. UV–Vis spectroscopy of the samples shows that the extinction in the near-infrared region is larger for the sample prepared with PVP (Fig. 3a). We also observe that the amplitude and the slope of the peak are large in the region from 500 to 800 nm, and that the amplitude is higher in the near-infrared region, suggesting a more complete shell. SEM images of the two samples confirm the effect of PVP on the formation mechanism of the shell described above (Fig. 3b,c). The particles prepared using PVP exhibit a higher surface coverage of gold. The gold is better distributed over the silica surface, resulting in a thinner and percolated shell, thus confirming the use of polymers as a powerful tool to fabricate thin nanoshells. On the contrary, nanoshells prepared without PVP exhibit isolated droplets on the silica surface (Fig. 3d,e). A shadowing effect in this microscopy image attests to sample charging, further evidence of a non-percolating shell (Fig. 3b).

**Figure 4:**
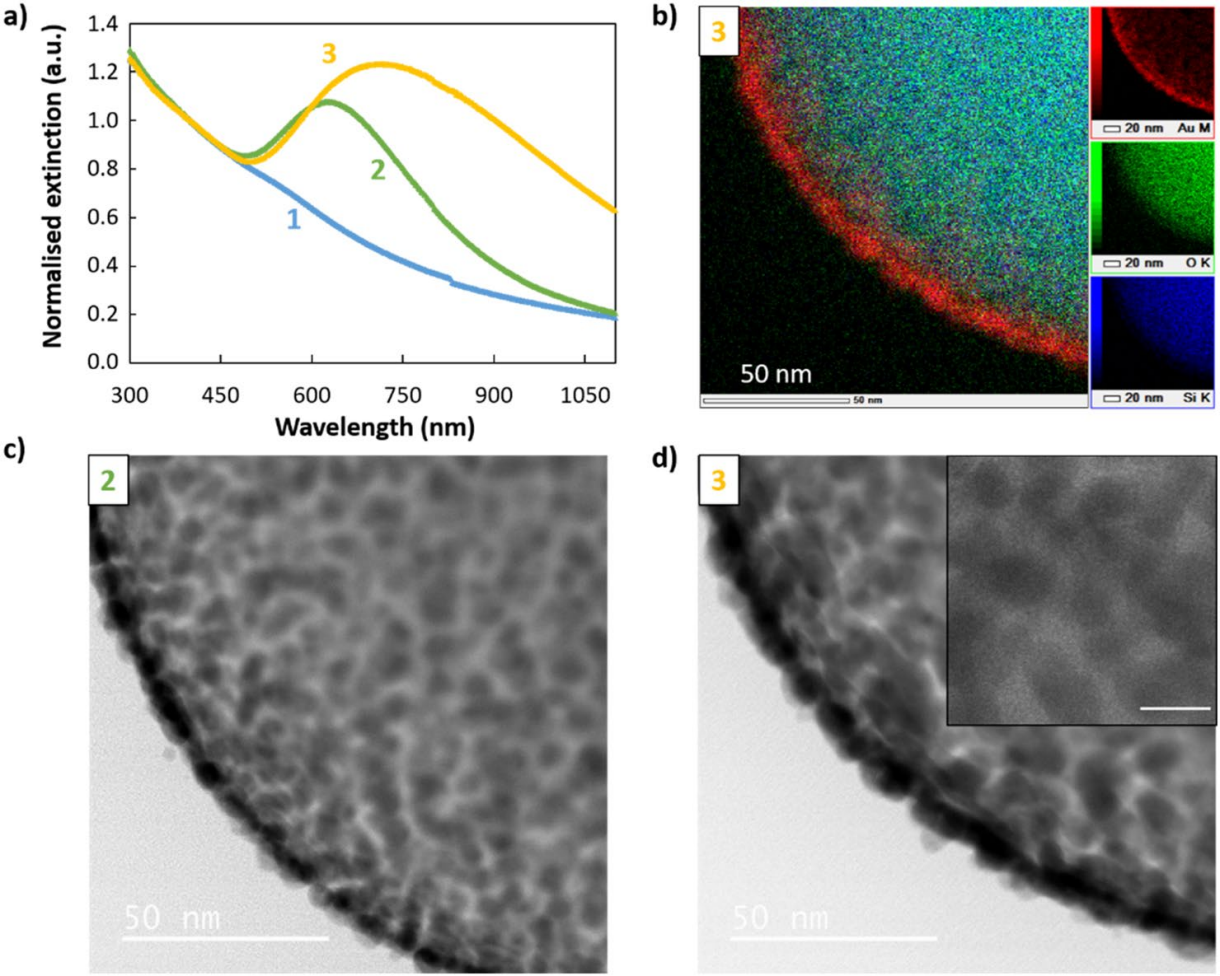
Characterisation of the gold shell growth synthesised in the presence of PVP. (**a**) UV–Vis spectroscopy of seed-deposited silica particles (1) before and (2 and 3) after regrowth of the gold seeds using a gold salt concentration of (2) 0.16 and (3) 0.32 mM. (**b**) TEM-EDX elemental analysis revealing (bottom right) silicon, (middle right) oxygen, (top right) gold, and (left) overlaid view of the core@shell sample 3. (**c**,**d**) TEM high magnification images of samples (**c**) 2 and (**d**) 3. Inset scale bar represents 10 nm.

To investigate the mechanism of the shell formation with PVP, we studied the particles at intermediate stages during the shell growth. The optical extinction of the particles during shell formation, in the presence of PVP, has a gradual appearance of a broad peak in the visible upon gold regrowth (Fig. 4a). The peaks are narrower and slightly blue-shifted compared to those of particles prepared without PVP and with the same gold quantity (samples 2–3 in Fig. 4a compared to green and purple curves of Fig. 2b). The high-resolution TEM images show that the shell growth, in the presence of PVP, forms first numerous tiny gold islands (sample 2 in Fig. 4c and Figures S3–S5) which grow and merge into larger, thin patches (sample 3 in Fig. 4b,d). At this stage, we estimate the shell thickness to be 6–7 nm based on both analysis of high-resolution electron microscopy images as well as EDX elemental analysis (Table S1). After functionalizing the surface with PVP and growing gold shells, even if the shell is rough (a few nanometers), we observe that nearly all the inter-seeds areas are filled as shown in the inset of Fig. 4d. Overall, it is clear that PVP favours a uniform seed growth and largely prevents dewetting. We attribute this to the following reasons: PVP forms a swollen polymer shell around the core–shell particle. For instance, a previous paper which used similar PVP concentrations measured a polymer thickness of 2 nm^32–36^. It possesses a strong affinity to gold (via C-N and C=O groups) and silica (from hydrogen bonding and from electrostatic interactions)^33,34^. Therefore, it is likely well adsorbed onto the seed surface, hindering particles from moving and thus preventing the Ostwald ripening process. This is consistent with the observation that multiple tiny islands occur in the presence of PVP, compared with larger islands in its absence. The presence of PVP may also shield the attracti attractive Van der Waals forces between the growing seeds, further hindering the coalescing process. We observe that the distance between the small tiny islands is much smaller than for large islands without PVP. For this reason, when the seeds are regrown, they need less additional Au to merge and form a continuous shell, allowing the fabrication of ultrathin shells. We want to mention two additional hypothesis that could explain the role of PVP in the improved continuity of the metal shell. First, a slower shell growth offers the possibility to match the reaction rate and the mixing to obtain a uniform shell growth, and therefore a thinner shell^23,31^. The reaction rate may be slowed due to the Au ions being complexed by the polymer. Second, it is possible that the PVP shell acts as a supramolecular architecture surrounding the core–shell particle. This could favor regrowth of the shell parallel to the silica particle surface as the growth perpendicular implies deformation of the shell which is energetically costly. Overall, PVP plays an important role during the growth process, improving both the smoothness and homogeneity of the obtained shells and enabling the synthesis of thinner shells. The approach presented here opens a simple way to obtain ultra-thin metal-coated silica colloids.

#### Post-synthesis microwave treatment

**Figure 5:**
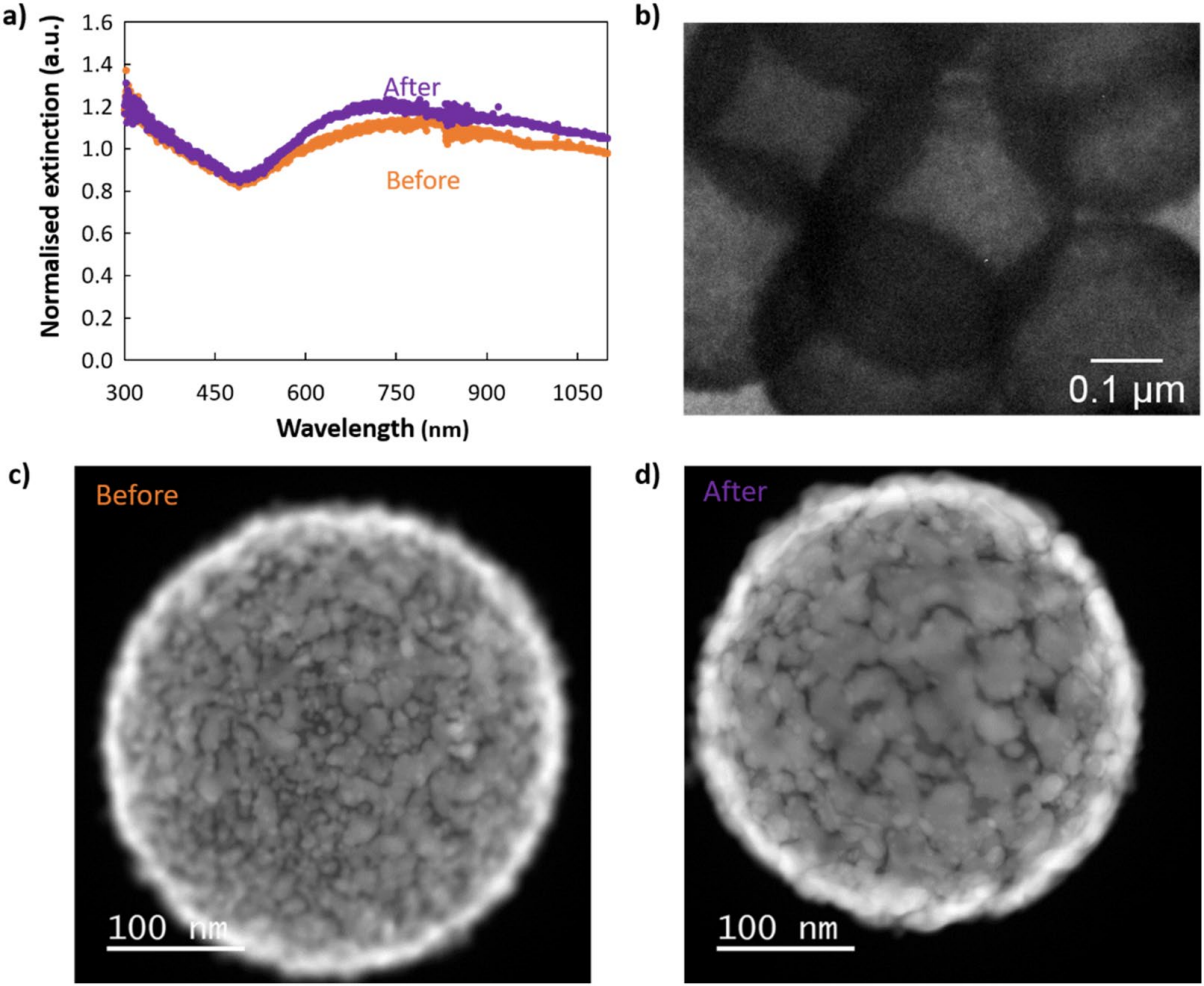
Flash microwave treatment of Au core@shell. (**a**) UV–Vis spectroscopy and TEM images of core@shell particles (**c**) before and (**b**–**d**) after a microwave treatment for 1 min at 140 °C.

Another approach to obtain a percolating shell consists of smoothing the nanoshell post-synthetically. To do so, we performed a thermal treatment of gold nanoshells (regrown from A_2_ without PVP) using microwaves. It simply consisted of a flash treatment, at 140 °C, for 1 min. Under other parameters (different microwave power and internal pressure), particle dewetting was observed (results not shown). Using these optimised conditions, improved optical properties were quickly and easily obtained. The UV–Vis spectroscopy shows that the treatment leads to a moderate increase in the extinction in the near-infrared region (Fig. 5a). This is consistent with small gold islands merging into larger and smoother patches, forming a more continuous shell (Fig. 5b–d and Fig. S6, Table S1). The nature of the effect of microwaves on this process is still unclear, as the interaction between the chemicals and strong electromagnetic field is complex and because several physical parameters are modified simultaneously: the local pressure and temperature as well as the global temperature. However, under appropriate settings, the temperature increase of the sample may occur so that the gold droplets soften without dewetting, leading to a not quite melted, smoother and thinner shell.

## Conclusions

Although the first synthesis of metallic nanoshells appeared 20 years ago, the synthesis of ultrathin shells with interesting extinction in the near-infrared remains challenging. We show that by combining a high ionic strength during the seed deposition with a double functionalisation/deposition step, we obtain a very high seed density on silica particles. However, we demonstrate that an increased seed density does not yield thinner shells, which we explain using the synthesis mechanism. In fact, seed regrowth is associated with the formation of less numerous, but larger droplets and their following regrowth eventually produces inhomogeneous nanoshells whose thickness is larger than 15 nm on average.

Once establishing that the limiting factor in obtaining ultrathin shells is not the seed-deposition, but the regrowth step, we turned our attention to this process. To achieve a more homogeneous seed regrowth, PVP was added prior to the gold plating solution. This widely used steric stabilising agent prevented seed dewetting into thicker shells, allowing a much more homogeneous coverage of smaller particles. We also employed a post-synthetic microwave treatment of the nanoshells to smooth the surface. By using a flash treatment, gold particle melting, which would inevitably lead to dewetting, was avoided, successfully producing a percolated shell. The combined modifications maintained precise control over morphology and our findings could also be extended to other metals to help improve the synthesis of ultrathin Ag or Cu nanoshells of high interest for energy saving applications.

## Methods

### Materials

l-Arginine (Sigma-Aldrich, 98.5%), tetraethoxysilane (TEOS, Sigma-Aldrich, 99%), ammonium hydroxide (NH_4_OH, J. T. Baker, 28–30% in water), sodium hydroxide (NaOH, Sigma-Aldrich, 98%), (3-aminopropyl)trimethoxysilane (APTMS, Sigma-Aldrich, 97%), sodium chloride (NaCl, Sigma-Aldrich, ≥ 99.0%), gold(III) chloride trihydrate (HAuCl_4_·3H_2_O, Sigma-Aldrich, 99.9%), polyvinylpyrrolidone (PVP, MW 44,000, Sigma-Aldrich, 98%), hydrochloric acid (HCl, Sigma-Aldrich, 37%), potassium carbonate (K_2_CO_3_, Alfa Aesar, 95%), ethanol (Sigma-Aldrich, 99%), formaldehyde (HCHO, Sigma-Aldrich, 37 wt. % in H_2_O, contains 10–15% methanol as stabilizer) and tetrakis(hydroxymethyl)phosphonium chloride (THPC, Sigma-Aldrich, 80%) were used as received.

### Synthesis

#### Silica particles

Silica particles were synthesised following the step-by-step published protocol^19^.

##### Synthesis of the silica seeds

An l-arginine aqueous solution (6 mM, 100 mL) was placed in a 150 mL double-walled vial, equipped with a reflux condenser. When the temperature stabilised at 60 °C, 10 mL of TEOS was added. The heating/stirring system was stopped when the TEOS upper phase disappeared.

##### First regrowth of the silica seeds

Ethanol (99%, 455 mL), ammonia (28%, 33 mL) and an aqueous dispersion of seeds (10 mL) were mixed and placed under stirring. TEOS (20 mL) was added to the solution with a syringe pump at the rate of 0.5 mL/h. Once finished, another 20 mL of TEOS was added using the same protocol. The as-obtained silica particles were approximately 125 nm in diameter. They were washed by two cycles of centrifugation/redispersion in ethanol (9000*g*, 10 min, 55 mL).

##### Second regrowth of the silica seeds

Ethanol (99%, 450 mL), ammonia (28%, 34 mL), water (10 mL) and the previous silica seed dispersion (5 mL) were added and stirred. Successively 20 mL, 15 mL and 8 mL of TEOS were added to the solution with a syringe pump at the rate of 0.5 mL/h. The synthesised particles were approximately 320 nm in diameter. They were purified by three cycles of centrifugation redispersion in ethanol (8000*g*, 10 min, 55 mL). The final volume of the silica nanoparticle dispersion was increased to 250 mL with ethanol and the silica concentration was measured to be 58.45 mg/mL by the dried extracts method.

#### Surface amination of the silica particles

The previous silica particle dispersion (8.55 mL corresponding to 0.5 g) was diluted in 200 mL of ethanol. APTMS (5 mL) was added to the mixture and stirred overnight. The dispersion was then heated to reflux overnight. The aminated silica nanoparticles were purified by three cycles of centrifugation/redispersion in ethanol (8000*g*, 10 min, 25 mL). Upon the final cycle, the particles were dispersed in 40 mL of ethanol with a final measured silica concentration of 19.5 mg/mL.

#### Gold seed deposition on the silica particle surface

##### Synthesis of gold seeds according to the Duff’s protocol^37^

Water (227.5 mL) was mixed with 0.2 M NaOH (7.5 mL) and 5 mL of diluted THPC (120 μL of the stock solution diluted in 10 mL of water) for 15 min. Then, 5 mL of HAuCl_4_ (25 mM) was quickly added and the solution immediately changed from colourless to brown, indicating the formation of gold seeds. The as-prepared gold-seed solution was kept at 4 °C for 14 days before use. Using an aged-solution of seeds rather than a fresh one for the deposition step favours the formation of silica particles uniformly covered with seeds^19^.

##### First deposition of the gold seeds onto the silica core (samples A_1_ and B_1_)

APTMS-functionalised silica nanoparticles (1.5 mL) were added to 38.5 mL of gold seed solution without NaCl (A_1_) or with an additional 3.478 mL of 1 M NaCl (B_1_). The dispersions were slowly agitated overnight. Excess gold seeds were removed by two cycles of centrifugation/redispersion in water (6000*g*, 10 min, 40 mL). After the final cycles, the particles were redispersed in water (10 mL).

##### Preparation of pre-polymerized APTMS solution

This synthesis was adapted from a published protocol^10^. Two volumes of 33 mL of water were mixed with 1.11 mL of HCl (0.1 M) and 1.33 mL of APTMS (stock solution) and vigorously stirred for 1 h.

##### Second deposition of the gold seeds onto the silica core (samples A_2_ and B_2_)

The two dispersions of gold-seeded silica cores (5 mL, samples A_1_ and B_1_) were mixed with the pre-polymerized APTMS solution (35.44 mL each) and stirred for 20 min. Then excess pre-polymerized APTMS was removed by three cycles of centrifugation/redispersion in water (6000*g*, 10 min, 40 mL). After the final cycle, the particles were redispersed in 20 mL of gold-seed solution without NaCl (sample A_2_) or with an additional 1.739 mL of 1 M NaCl (sample B_2_). The dispersions were slowly stirred overnight. The excess gold seeds were removed by two cycles of centrifugation/redispersion in water (6000*g*, 10 min, 40 mL). After the final cycle, the particles were redispersed in water (5 mL).

#### Formation of the thin metallic shell by a controlled gold seed growth

##### Preparation of the gold plating solution

50 mL of an aqueous solution containing 4 mL of HAuCl_4_ (25 mM) and 125 mg of K_2_CO_3_ were prepared and stirred for 3 h. A colour change from yellow to colourless was observed. The gold plating solution was stored in the dark at 4 °C for 24 h.

##### Gold seed growth

The gold-seeded silica particles (250 µL, A_1_, A_2_, B_1_ and B_2_) were mixed with water and with different volumes of gold plating solution and formaldehyde. Water was first added so that the final volume of the solution was 10 mL. For the experiments displayed in Fig. 2a–d, the gold plating solution volumes used were 100, 800, 1600, and 2666 µL, respectively. The volume of formaldehyde added was 1/20 of the gold plating solution volume, with a maximum of 40 µL of formaldehyde. The colour change occurred within a few minutes. The excess reactants were removed by two cycles of centrifugation/redispersion in water (from 1000 to 4000*g* depending on shell thickness, 5 min, 10 mL). After the final cycles, the particles were redispersed in water (10 mL).

##### Gold seed growth in presence of polyvinylpyrrolidone

The previous protocol was carried out except that an aqueous solution of PVP (1 mL, 10 g/L) was added before the gold plating solution. The colour change occurred over a few hours instead of minutes. After the final centrifugation, the solutions were redispersed in PVP solution (10 mL, 1 g/L).

#### Microwave treatment

An Anton Paar microwave reactor (Monowave 450) equipped with an autosampler (MAS 24) was used. The as-synthesised particles (2 mL), regrown from A_2_ without PVP, were added to water (18 mL) in a 30 mL vial. The solution was heated with microwave radiation to 140 °C for 1 min.

### Characterisation

#### Electron microscopy characterisation

Scanning transmission electron microscopy (STEM) studies were performed using a JEOL cold-FEG JEM-ARM200F operating at 200 kV equipped with a probe Cs corrector reaching a spatial resolution of 0.078 nm. EDX spectra were recorded on a JEOL CENTURIO SDD detector. For Transmission electron microscopy (TEM) experiments, samples were prepared by depositing one drop of the colloidal dispersion onto a conventional carbon-coated copper grid. Grids were air dried at room temperature and stored in a closed box to prevent dust accumulating. TEM experiments were performed using a JEOL JEM 1400+ operating at 120 kV with a LaB6 filament. Scanning electron microscopy (SEM) experiments were performed on a SEM-FEG HR—JEOL 6700F. Shell thickness is estimated over a limited number of particles by analyzing high-resolution images obtained by HRTEM and STEM-EDX elemental analysis. We used images zoomed-in on the particle edges, where we can differentiate the silica particle and the metallic shell.

#### Optical characterisation

UV–Vis spectroscopy spectra were recorded on a Shimadzu UV-3600 UV–VIS-NIR.

## Supplementary Information

Supplementary Information.
