# Supplementary material for: Seeded growth of ultrathin gold nanoshells using polymer additives and microwave radiation
Source: Sci Rep. 2021 Sep 8;11:17831. doi: 10.1038/s41598-021-97171-0 (PMC8426366; doi:10.1038/s41598-021-97171-0)
Supplement: Supplementary file 1 — Supplementary Information. [file 41598_2021_97171_MOESM1_ESM.docx]

Supporting information

Seeded growth of ultrathin gold nanoshells using polymer additives and microwave radiation

Laurent Lermusiaux^1,^*, Marie Plissonneau^2^, Laure Bertry^2^, Glenna L. Drisko^1^, Valérie Buissette^2^, Thierry Le Mercier^2^, Etienne Duguet^1^, and Mona Tréguer-Delapierre^1,^*

^1^Univ. Bordeaux, CNRS, Bordeaux INP, ICMCB, UMR 5026, Pessac 33600, France

^2^Solvay R&I, 52 rue de la Haie Coq, 93306, Aubervilliers, France

This section contains supplemental figures including TEM and HRTEM images of the core@shell particles synthesised at different conditions as well as a table summarizing the characteristics of the different samples.


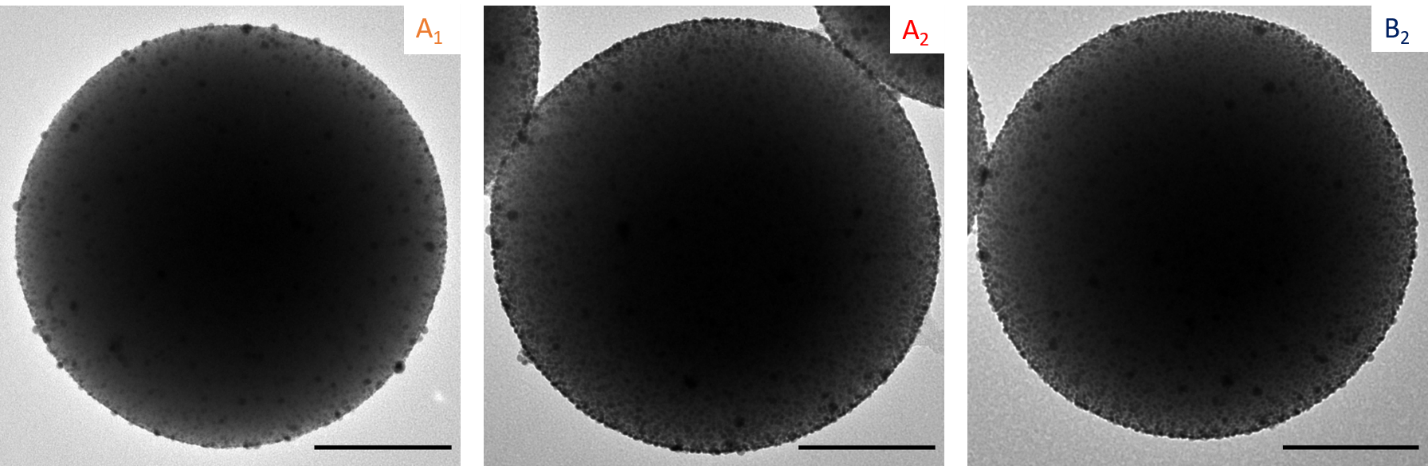


**Figure S1.** Transmission electron micrographs of functionalised silica covered with different gold seed densities, labelled as in Figure 1. Scale bars represent 100 nm.

**Table S1**. Estimation of the continuity, thickness and smoothness of different nanoshells, using the following parameters respectively: shell coverage, average shell thickness and its standard deviation.

| Figure | Sample characteristics | Shell coverage (Approx.) | Average shell thickness (nm) | Standard deviation (nm) |
| --- | --- | --- | --- | --- |
| 2e | No PVP, after shell regrowth | 90% | 13.6 | 2.2 |
| 4d | PVP during regrowth | 95% | 6.2 | 1.2 |
| 5c | No PVP, before microwave | 90% | 13.4 | 1.6 |
| 5d | No PVP, after microwave | > 98% | 13.0 | 0.9 |


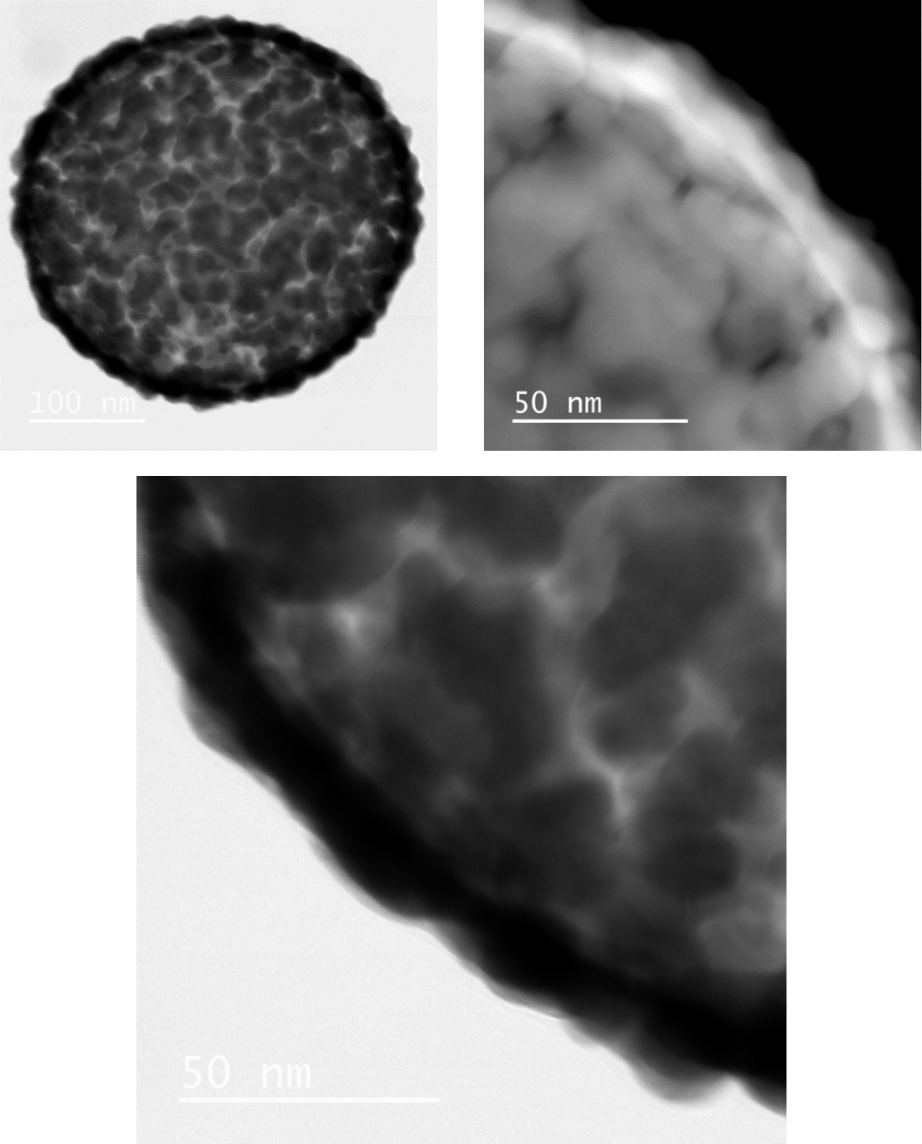


**Figure S2.** TEM images of the core@shell particles, synthesised without PVP, corresponding to Figure 2e. The average shell thickness is about 13-14 nm.


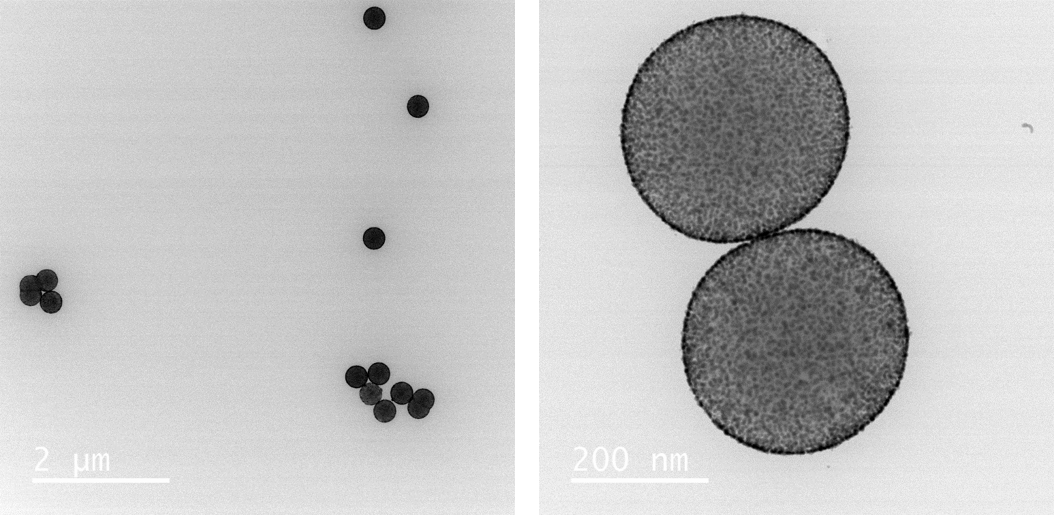


**Figure S3.** TEM images of the core@shell particles, synthesised with PVP, corresponding to the sample 2 on Figure 4c.


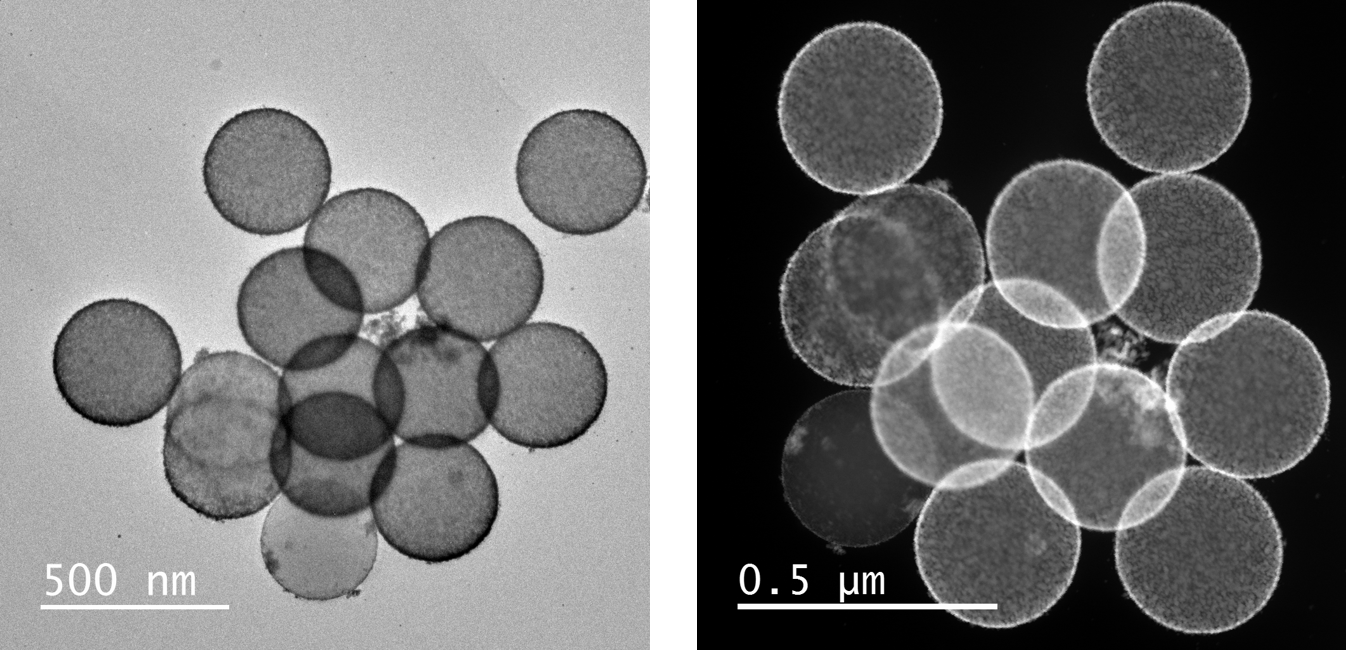


**Figure S4.** TEM images of the particles with ultrathin shell, corresponding to sample 3 on Figure 4c.


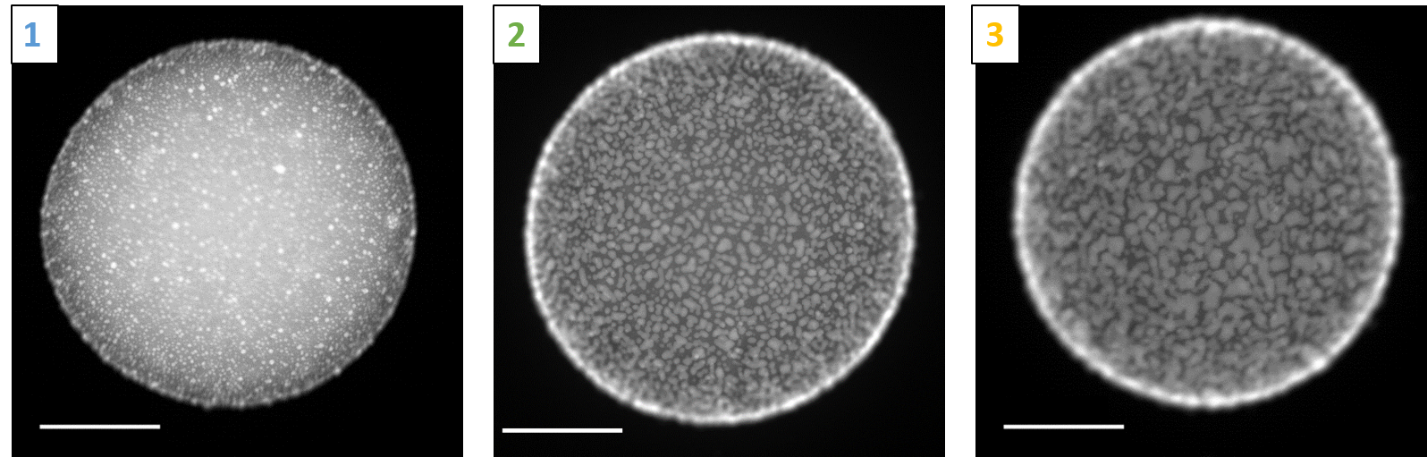


**Figure S5.** TEM high magnification images of the core@shell synthesized with PVP at different intermediate stages during the shell growth. The PVP favors the growth parallel to the silica surface. Scale bars represent 100 nm.


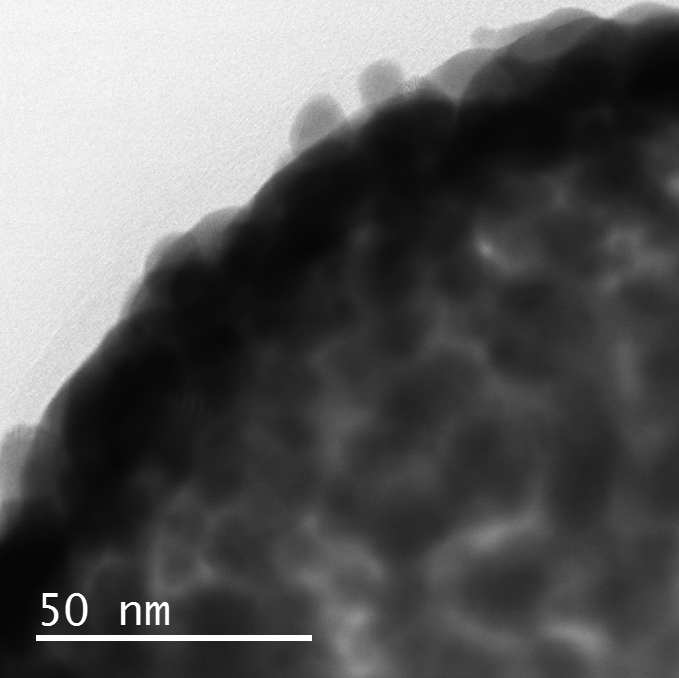

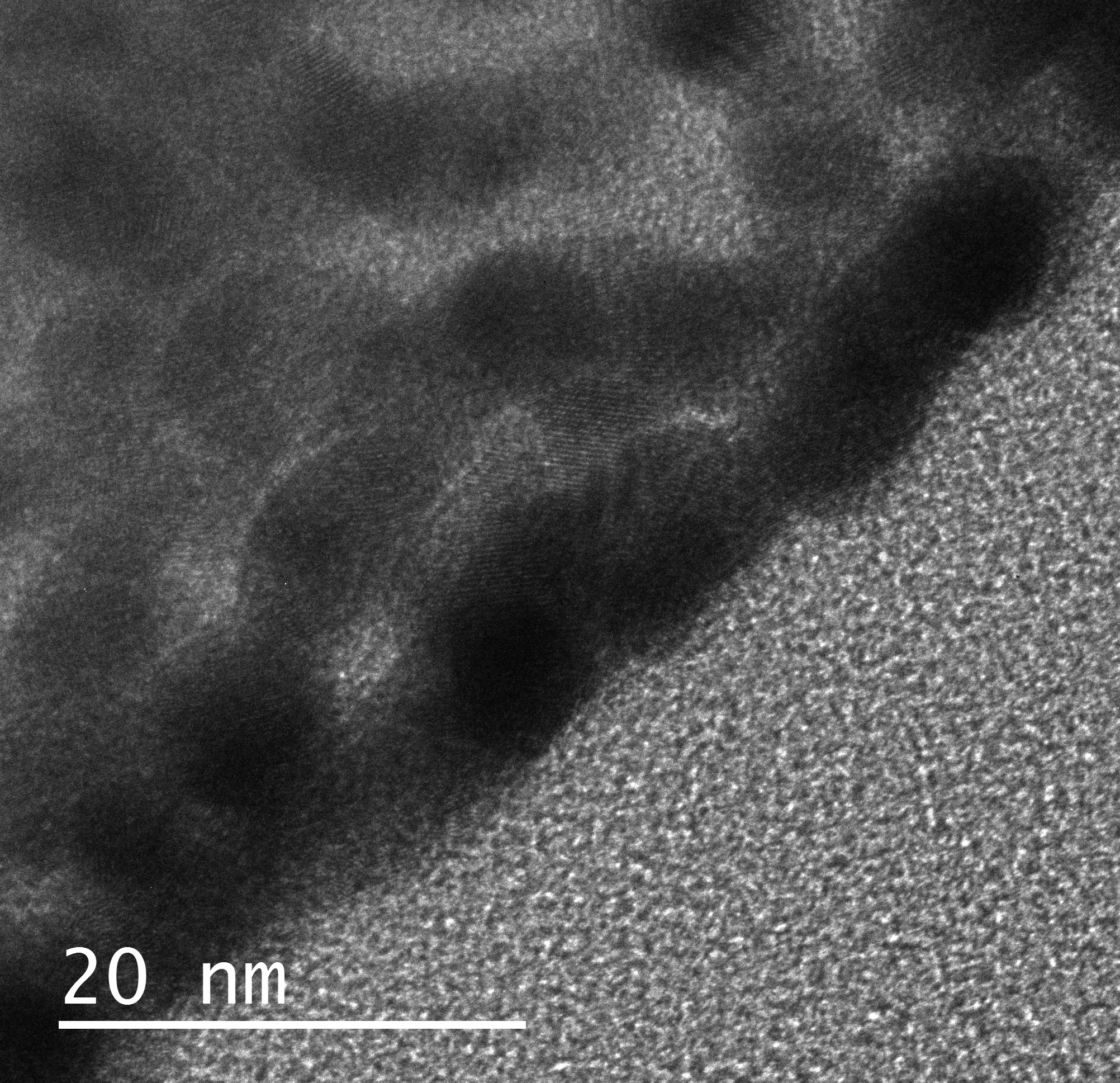


**Figure S6.** High magnification image of the core@shell particles synthesized after a microwave treatment of 1 min at 140°C.
